# Translational health technology and system schemes: enhancing the dynamics of health informatics

**DOI:** 10.1007/s13755-020-00133-5

**Published:** 2020-11-09

**Authors:** Marjo Rissanen

**Affiliations:** School of Science, Aalto University, Espoo, Finland

**Keywords:** Health technology, System dynamics, Translational design

## Abstract

Translational health technology and design schemes reflect certain themes in systems approach and its dynamics. This paper discusses these aligned ideas in view of their value to translational design processes. The ideas embedded in these two approaches are considered in the light of critical questions associated with the development of health informatics. Health care processes for patients might be very fragmented. Synergy thinking is required in all areas of design: it is crucial to understand the theoretical frames and issues associated with focus environments, administration, and cost policy. By internalizing common nuances in these approaches, designers can ease the interaction and communication between experts from different backgrounds. Synergistic thinking aids designers in health informatics to produce more sophisticated products. Maturing in recognizing the whole aids to take into account “the very essentials” more easily. These skills are very vital in prioritizing development substances in health informatics area.

## Introduction

Increased specialization in healthcare requires concentration and this specialization need grows all the time partially because of the needs created by new technologies. Nowadays we have more data, evidence-based guidelines, and simplified rules-based algorithms to aid in decision-making but the amount and complexity of options may decrease abilities to synthesize it [1, 2]. Modern complex software systems require also more advanced data mining techniques because healthcare is increasingly relying and investing on them [3].

Therefore it is essential to manage and understand the “big picture” and the formation of the new entities from the known sections. Interoperability is more than systems exchanging information because it includes also technical, human, and educational aspects [2]. Because of the vivid production intensity in digital scheme, also an efficient coordination policy is needed to evaluate what kind of system integration serves best intensity improvement in healthcare and means at the same time reasonable resource use and optimization of human and machine power. Care coordination failures represent a remarkable waste domain example in healthcare [4]. All kinds of developments needs in healthcare concern cost policy. Understanding the entities is in this respect one essential part of translational design challenge. Internalizing this encounter is an interesting research topic and motivates to focus on it.

This research discusses the ideas described in systems approach (part 2), particularly their connections to the aims and challenges of translational health informatics (part 3). The utility value and limitations of systems approach in translational health technology projects are also explored through contextual literature and the concept behind the analytical approach (parts 4, 5).

## Systems thinking and structures in the context of translational design

System structures and dynamics provide insights that are recognizable in health-related translational design. The purpose of translational health informatics is to deliver high-quality products with positive environmental, social, and ethical impacts. Translational research is defined as “the process of applying ideas and discoveries generated through basic scientific inquiry to the treatment or prevention of human disease” [5]. In this context, health informatics plays a remarkable supporting role when targeting cost-effective prevention and treatment strategies. Well-established supporting models are often advantageous for design practices and the implementation phase and to prepare beneficial learning contexts for the public and private sectors, organizations, and corporations [6]. Synergistic thinking is needed in health informatics because integration requirements grow in spite of fragmented design and clinical practices.

Systems thinking and its applications have yielded useful results in many practical situations by serving as a bridge between theory and practice [7]. The rules for applying systems thinking include “questioning system boundaries, system structure and interrelationships, adopting multiple perspectives, considering dynamic characteristics and applying a holistic, big picture view” [7]. Systems thinking itself has been interpreted in various ways, and differences persist regarding its definition and understanding [7]. The systems thinking literature on systems engineering includes theoretical, methodological, and practical methods for applying systems in various disciplines and areas [7].

According to Meadows [8], “system dynamics is a set of techniques for thinking and computer modeling that helps its practitioners begin to understand complex systems...System tools help us keep track of multiple interconnections; they help us see things whole.” System dynamics has a solid role in science and engineering as well as education and real-life applications when attempting to understand organizations, systems, procedures, and related interactions [9, 10]. A sociotechnical system involves ethical, social, organizational, and technological dimensions and is thus a concept regarded as significant in the eHealth area [11, 12]. Critical areas of translational medicine have been examined from the perspective of systems thinking [e.g., [13]]. Systems thinking provides opportunities for understanding how human health may be improved, and related tools have been used successfully in health care [14–16]. The current areas of interest in translational medicine include health systems research [17]. Systems thinking appears to help policy partners reconceptualize health problems and change thinking patterns in preventive medicine [18]. Design science research on information systems involves a wide range of socio-technical artifacts, such as decision support systems, modeling tools, strategies, and methods [19]. Systems thinking in design practice is thought to suffer from some restrictions; specifically, a clear identification of the problems designers might address can lack [20]. From the design perspective, at times, it is best to understand a system as an organic whole of units and elements instead of an assembly or arrangement of parts aggregating the whole [20].

## Translational design challenges and involvement of system dynamics

### Managing complexity by decreasing complicatedness

In systems approach, “simple ideas lead to simple models that can serve as elements of more complex ones and thus models of complex systems are obtained by combining relatively simple building blocks” [9]. Health information systems are complex entities, wherein the whole should comprise a combination of integrative smaller units. This complexity grows continually with systems enriched by new kinds of functions. However, complexity is also regarded as an inherent property of a system in terms of its architected complexity, which can actually reduce the complicatedness of the system [21].

In customer service, a personal health record, for example, may involve connected encounters for health management, information delivery, consumer feedback, and contact creation. The challenges in health informatics are associated with not just proper integration and interoperability but also enough “simple blocks.” These blocks should represent systems and applications in which the mission of each module is justified. Systems should also possess deep clarity to efficiently serve health professionals and consumers. Clarity has an essential role of ensuring the safety of the electronic health records (EHR), connected encounters, and functionalities (e.g., systems for learning, clinical documentation, decision support, etc.). In addition, health informatics should also be equipped with the “quick impression” characteristic [22], which means that an informative message is quickly detectable and understandable. This characteristic plays a critical role in realizing safe and efficient clinical practice. The more versatile and feature rich the application is, the more significant is its reduced visual appearance and compact functional state so that it would not increase cognitive burden and thus act as a safety hazard in clinical practice. Information technology and systems in healthcare should actually serve as change agents in streamlining service processes [23].

### Concept of the “whole” in system development

As “science and engineering have become complex and divided,” system dynamics has emphasized the ability to see particles as a whole again by helping identify interconnections [9]. This idea is aligned with translational targets; the aim being to create interaction and synergy between medical disciplines and practices via increased data integration and interoperability [24]. A distributed system also ”appears to its users as a single coherent system” [25].

The holistic approach in health informatics refers to the ability of the designer to view individual contributions as a meaningful part of the whole. Added pieces and sub-systems should be seamless parts of the entire system with their added value in spite of the fragmented design procedures and distributed systems. Similar to the design process, patients’ care processes can also be very fragmented. One purpose of consumer-targeted systems is to minimize this threat with proper coordination. In integrative design strategy, the entire service chain (i.e., applications, services, consumers, and health professionals) should form a fluent and seamless whole in spite of fragmented clinical practice and design processes.

Theoretical frames guide designers in their work. Typically, designers’ attention in the development process is focused mainly on product quality. A versatile framing of quality could allow designers increased capacity in the production process by helping identify connections between different quality views [26]. This also aids designers in understanding the logic of adoption and dissemination policies in health care [27]. The visual dimension of applications e.g., is easily perceived primarily as an aesthetic feature, but this aspect has much to do with the usability, safety, and functionality of applications [28]. A profound understanding of the interconnections of different frames provides a brighter vision of the whole in design practice.

### Enhancing health informatics with creativity

Creativity and intuition are emphasized in systems thinking and translational health policy. Design is regarded as a search process that involves creativity, innovation, and intuition because the existing knowledge base is often insufficient for design purposes [29]. A continuing dialogue is essential between systems thinking, creativity, and other areas and disciplines [30]. As known, not all problems are solvable in a stepwise fashion or by considering interdependencies [31]. The general analysis–synthesis— evaluation process does not fit design, because “design initiates novel forms which require more intuitive ways of thinking and reasoning” [32]. It is argued that system analysis does not reveal the problems that could be addressed by human action to change compound situations [20, 33]. Moreover, problems are found via concrete experiences and circumstances rather than through the identification of elements that create the complexity [20, 33].

Creative design requires in healthcare a profound understanding of the known and hidden problems in the domain area and its system environment. Simultaneously, it should possess the ability to “forget” existing practice models and protocols. Therefore, besides proactive thinking and interdisciplinary work [6], the health sector requires domain-conscious understanding with a flexible mindset. Profound substance knowledge is favorable when trying to increase cost-efficiency while applying innovations in health informatics. Creativity and intuitive thinking often go hand-in-hand. Intuition as felt knowledge—although often overlooked today—improves decision making in organized or less-organized situations [34, 35]. Novel solutions must, naturally, be fit for context domains [36]. When intuitive design is connected with domain-conscious professionalism, it is not out of the question in the health sector.

### Cost policy and the need to understand the “whole”

Health informatics is considered as an important tool in managing cost-efficiency and outcome quality [37]. The increased push to control expenditures has led to a phenomenon wherein savings are done in critical areas of clinical practice, which actually play a minor role in the overall cost policy. Random reductions of laboratory budgets is one such example [38]. Laboratory costs constitute approximately 3% of all clinical costs [38]. Correspondingly, diagnostic errors contribute even 70% of all medical errors [39]. Improper testing leads easily to misdiagnosis and inappropriate care. Therefore adequate laboratory testing has a remarkable role in diagnostics. [1, 4] Diagnostic errors are the most hazardous, costly and common type of medical mistake and are most likely to result in disability and death [40]. According to estimates medical errors are the third-leading cause of death after heart diseases and cancer in US [41]. In that light AI (artificial intelligence) represents considerable help in preventing, in part, human error [42] but does not prevent necessarily all harmful managerial decisions. In the ambulatory setting, in 59% of the diagnostics related malpractice claims 30% resulted in death [43]. Random reductions of laboratory budgets can risk quality of care and increase total costs [38]. Diagnostic errors represent key elements among the major contributors to wasteful spending [1, 4].

**Fig. 1:**
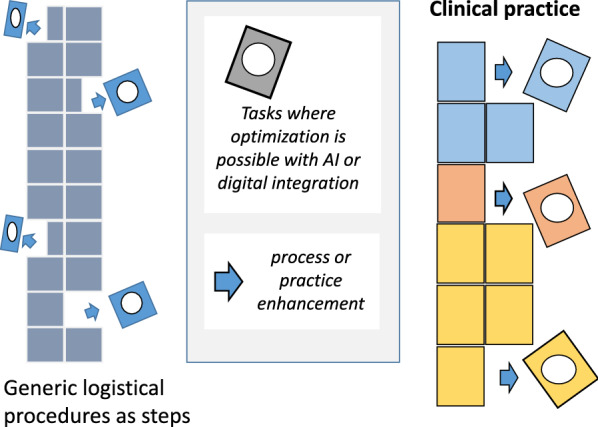
Resource optimization with AI and digital integration in clinical practice

On the other hand, personnel costs are known to form the majority of total costs in hospitals. In prudent design, health informatics can offer a potential for real savings. Examples and scenarios show how digital services and advanced machine learning algorithms with proper logistics offer possibilities to forecast, optimize, and rationalize the resource and time usage of physicians and other health professionals through new solutions and protocols [1, 42, 44, 45]. AI tools are also helpful in patient flow optimization and in repetitive processes [42]. Tailored treatment algorithms would reduce inappropriate care and maximize favorable patient outcomes [1]. But the question should be also about health consumers’ resource use. Clients may still sit for hours in the waiting room of hospitals instead of waiting for notification on their mobile phone of an estimated consultancy time. Innovations in health informatics can change health care structures radically and in a cost-effective manner if this potential is used rationally, giving space for new, however ethically justified, insights (in Fig. 1).

### Whole in AI implementation

Forms of Artificial Intelligence (AI) like deep learning algorithms and neural networks are explored for novel healthcare applications in areas like imaging and diagnostics, treatment, risk analysis, health information management, virtual assistance, and patient monitoring [45, 46]. In addition to advanced mathematical models, the implementation of AI can be perceived as AI-based software that informs or influences clinical or administrative decisions and healthcare delivery [47]. Big data, data integration, and combined information allow physicians in discovering more consistent patterns of diseases. In Europe researchers found five distinct types of diabetes instead of two by analyzing data of 14 755 patients. A refined classification is useful in individualized treatment and better identification of patients with increased risk. [48]

Cardiac arrhythmia causes approximately 12% of all deaths globally. Medical Internet of Things (IoT) means many new opportunities to health care. IoT-based devices are more human-friendly because of smaller, compact size. IoT-based ECGs e.g., have brought new ways for the automated cardiac arrhythmia detection and follow-up. In this area a new deep-learning based innovation which consists of two modules: a data cleaning module and a heartbeat classification module assists in heartbeats identification and classification making these innovations even more reliable. [49]

Alzheimer’s disease is the most common form of dementia and a major health problem. Mild cognitive impairment (MCI) can reveal the early stage of this disease and electroencephalography (EEG) is a good choice in detecting of MCI biomarkers. A model which is based on modern machine learning technique (Extreme learning machine, ELM) has been used successfully to distinguish MCI from healthy control subjects with good classification accuracy (98,78%) and fast processing time. [50]

Assessment of vascular characteristics plays an interesting role in many medical illnesses, like diabetes, hypertension, and arteriosclerosis. Retinal vascular disorders refer to many eye diseases. Machine-based quantification of retinal vessels assists ophthalmologists in screening processes. In combined approach is used colour coded texture mapping that increased accuracy in image analysis of the retina and automates the analysis of retinal vessel widths. [51]

AI algorithms have also a role in medical imaging e.g., for breast cancer in improving the performance of mammography and in diagnosing breast cancer at a higher rate than pathologists [42, 52]. Pollen allergy is a global concern. Neural networks model combined with data and local social media is utilized successfully in the classification of allergy symptoms. [53] In the field of dermatology AI has the potential to assist in the diagnostics and at the interface between primary and secondary care [54]. AI applications serve in clinical practice also in pathology and by robot-assisted surgery, in precision robotic treatment, and in virtual reality-enabled robotics [42, 45]. As well, AI has a potential role in biopharmaceutical development, in cloud –based digital drug discovery, and in deep genomics in clinical trials [42].

However, it is remarked that promising technologies are not reaching the patients and healthcare systems because beneficial products are not deployed at the rates required [55]. Despite the promise of machine learning the availability of sufficient high-quality data means limitations as well as the interpretability of machine learning algorithm output [1]. Also clinical research strategies for systematic AI evaluation lack [47]. Such evaluation should not focus only on the technical properties of AI but moreover on the challenges of using AI in clinical practice [47]. Fulfilling the potential of AI: “better care at lower costs” requires AI-related best practices and understanding connected ethical challenges as well [46, 55].

### Teams as creative systems

Teams can be regarded as systems [10]. Multidisciplinary teams are required in translational science to contribute to the idea of the whole [56]. Synergistic thinking, interaction, and diverse perspectives are emphasized in translational strategies [9]. The importance of the multidisciplinary approach and team synergy is also emphasized in systems approach [57]. As known, often new perspectives and enhanced heterogeneity can increase competence [10]. The greatest challenge, however, lies in bringing together researchers and practitioners from various specialty areas [58]. Moreover, current health care systems do not always promote team synergy [6].

Despite the increasing number of innovations in health technology, many of them were implemented prematurely without sufficient evaluation, resulting in wastage [59]. Therefore, effective and versatile team effort is needed in every design stage [59]. Interdisciplinary research is helpful for innovation, but it is also needed for the application of ideas in practice [6]. Health consumers are perceived as team members and co-designers in health informatics. Conversely, gathering user experience and consumers’ ideas requires more than flexible channels for user feedback. This engagement challenge is especially notable in elderly care.

**Fig. 2:**
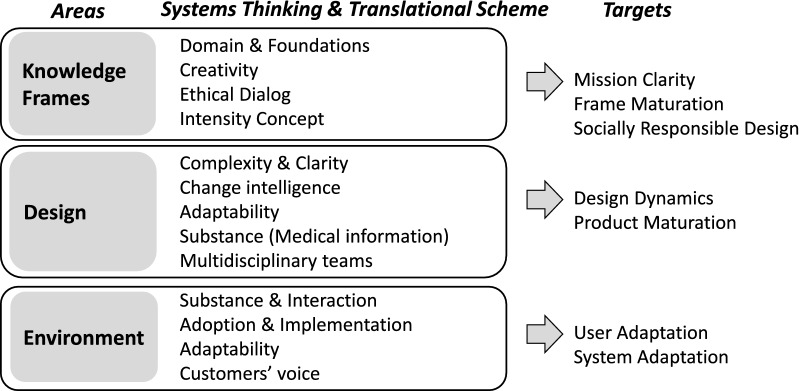
Synergy thinking in design. The structure of areas follows Hevner et al. [29]

Team construction policy in health care requires an unprejudiced mindset. Novel innovations and paths may necessitate special know-how associated with new areas. Different specialists, however, need to become familiar to a certain degree with domain-specific know-how, frames, and problematics. This requirement in the health sector is characterized by the designer’s ability to “understand and adapt to the language of the domain rather than to the language of the design and user experience” [60]. Therefore, besides domain experts, the health sector needs designers with a versatile understanding of the environmental components in strengthening various design practices. The synthesis of knowledge is one of the keys to achieving more adaptable design (Fig. 2).

### Understanding the ethical whole

Despite low levels of engagement between systems scientists and health professionals (e.g., in bioethics and public health ethics), such debates are viewed as useful [61]. Translational health policy also requires the integration of ethical tools [62]. New approaches in translational research ethics are needed to advance research in this field [62]. Designers in health informatics require a versatile understanding of ethical quality. For instance, ethical quality connects profoundly the other attributes of quality, such as products, customers, and processes [26]. The system’s role and capability as a real process intensifier is an evaluation question with ethical emphasis, because the question is also about the justification of investments. The ethical acceptability of a product should be of primary concern in the pre-design phase of translational eHealth projects.

Health information privacy, industry relationships, bias and accountability for machine error are timely ethical questions [1, 63]. Privacy worries e.g., are the main reason for the low adoption rate of personal electronic health records [64]. On the other hand, in the design context of health informatics, ethical quality is easily perceived as having too narrow a scope (e.g., mainly as a matter of information security and privacy). When the role of ethics is internalized in a versatile way, its synergetic nature can be perceived by designers, and intensive, socially responsible design becomes a real possibility.

### When the sum is greater than the parts in the learning experience?

**Fig. 3:**
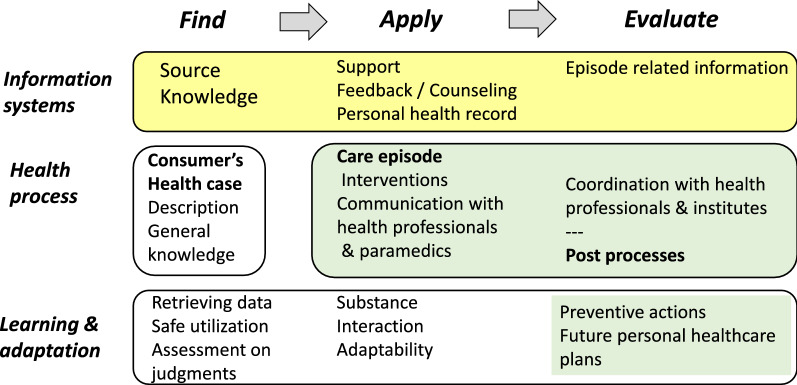
Synergic learning environment in health care

Systems thinking is considered as an iterative learning process that directs the practitioner toward holistic and dynamic views [65]. Cumulative knowledge has contributed to systems sciences and has played a role in many areas of translational research [66, 67]. In healthcare, information systems form only one subsystem of a consumer’s learning environment. Patients’ health care episodes and all connected interventions and engagement with health professionals form the knowledge entity as well as the supportive information systems, which aid consumers in different phases with regard to their knowledge needs. This entity denotes the whole in the learning contexts of patients (Fig. 3).

In the synergetic learning model the purpose is that “adding any component to a learning solution increases value”; resulting the sum, greater, than the individual parts [68]. The intention here is to allow health information systems to streamline the synergy of the learning entity, such as by supporting the coordination between phases. Designers should consider the value of modules in view of their role as coordination supporters for the whole care episode. At times, vivid production intensity creates situations in which patients or consumers are offered several and somehow overlapping, products that frustrate them. Coordinative actions represent the most demanding specialty area in clinical practice. The same task applies to health informatics policy. Health informatics has a considerable role in enhancing the coordination of patient care and treatment. This is essential because care coordination failures represent a significant waste domain in health care [1].

### Process synergy and intensity issues

Health care processes can be divided into various phases and different units. The vision of the whole disappears easily because of the fragmented nature of the care process, which also weakens the care intensity level and its evaluation. Poor coordination increases risks also in clinical practice in healthcare [1, 69]. Care intensity evaluation throughout the process contributes to the synergy of care processes. Patient satisfaction does not always denote better outcomes [70]. However, higher care intensity is often connected to better outcomes, lower mortality, and increased satisfaction [71–75]. Moreover, higher care intensity may be attained without significant cost difference e.g., in intensive care and also with favorable clinical outcomes among critically ill elderly patients, even if older age is often associated with less intensive treatment [74, 76, 77]. Patients recognize several problems that are not identified by common reporting systems [78]. However, patients also feel that they do not have enough influence over their care processes [79].

Patient feedback plays a valuable role in creating coordination and synergy between the provider and consumer. The majority of patients’ feedback issues are related to non-satisfactory care intensity levels; common complaints involve disagreements over treatment, insufficient information or lack of communication, lack of confidence, unavailability of physicians or other health staff, and negative front-desk experiences [42, 80–83]. Low-quality care and care delivery failures are remarkable contributors to wasteful healthcare [1, 4].

Best practices must be implemented to ensure safety, obtain feedback at all levels of health care, and facilitate the effective use of such systems [84, 85]. Besides various feedback channels, systems that provide just-in-time responses to the given feedback are essential [86, 87]. To address the shortcomings identified, improving intensity in the form of well-functioning feedback and engagement systems deserves more attention in health policy and design of quality strategies. Feedback systems that target care intensity evaluation should not represent only one mandatory step in a procedure. “Hearing the customers’ voice” means that the evaluative feedback of patients on perceived care intensity leads to necessary actions, which will also be evaluated in turn. AI is a promising tool in producing needed feedback information for wider comprehension [42]. When trying to benefit from AI, these possibilities should be taken into account already in the design phases of more functional and user-friendly feedback systems. Collecting, curating, and labeling data is expensive and laborious [1]. In advanced data mining processes first is needed a novel idea that allows identification of the key components and then their composing into smaller independent ones in discovering component based models [3]. Likewise, ideas on how to get the most out of the feedback are needed first. What are the key components necessary to know in genuine enhancement aims? How these should be decomposed into smaller independencies in order to produce big data beneficial for patients and organizations?

**Fig. 4:**
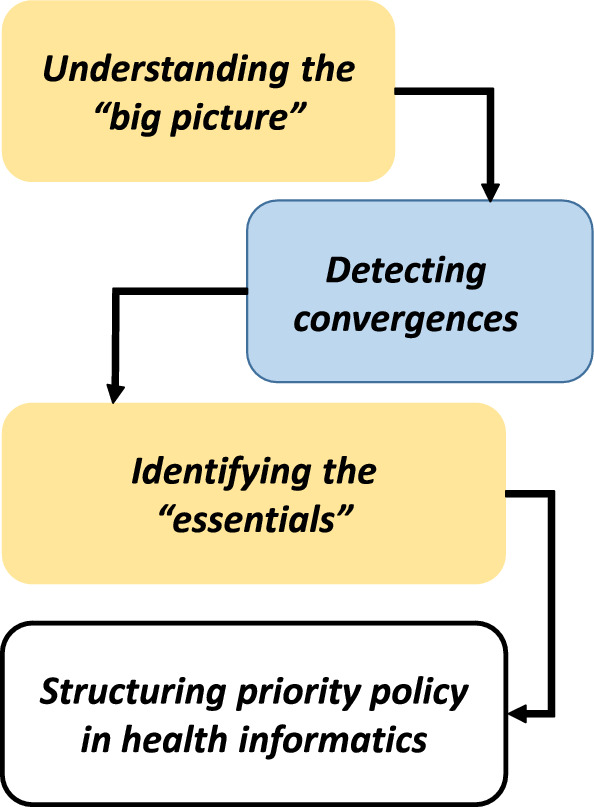
Synergy thinking as an aid in priority policy

## Discussion

Observing ”the holistic big picture” is crucial in daily clinical practice and in all advance in medical field. Comprehending entities more properly also aids to identify the convergences and thereafter “the very essentials” more easily. These skills are very vital in prioritizing development objects in health informatics area (see Fig. 4). Big data, as is, is useless without proper analyzing tool and knowledge [88], and first of all, without eagerness to utilize it successfully with awareness of connective ethical and legal aspects. Diverse expertise and smart combinations are often crucial in significant advances. At this stage there is a lively debate on AI liability issues. In particular the so called algorithms of unknown chains of reasoning (black box phenomenon) are seen as problematic [89]. In addition to shared responsibility, emphasis is also placed on the doctor’s overall interpretive and evaluative responsibility in situations. However, despite the complexities clarifying liability issues is important for the realization of patients’ legal protection [90].

AI is changing the cost thinking perspective in healthcare in a meaningful way as it contributes to shifting attention to a more significant cost area. While AI creates possibilities to enhance the intensity level of care it creates opportunities for optimizing human resources as well. The direction is right as staff costs are the most significant cost item in hospitals. When savings are tried to achieve by random reductions e.g., in areas of diagnostics and treatment an adequate care intensity level may suffer. Compromising the appropriate care intensity level can ultimately be extremely costly with possible fatal consequences. However, AI systems are not expected to completely replace or automate human resources [90]; it is mainly about adjusting staff resources. As stated, the question is about prime synergies, new openings for restructuring and thus potential savings. However, this streamlining and optimizing the performance of tasks between man and machine does not happen by itself only by exploiting the potential of AI. It also requires the design of operating models and processes from a different perspective. AI should be seen in healthcare as an aid to action and better rationalization. AI is a matter of synergy and the search for appropriate synergy models. When optimal combinations produce in health care cost savings with wanted health outcomes, this means synergy savings and synergy benefits.

**Fig. 5:**
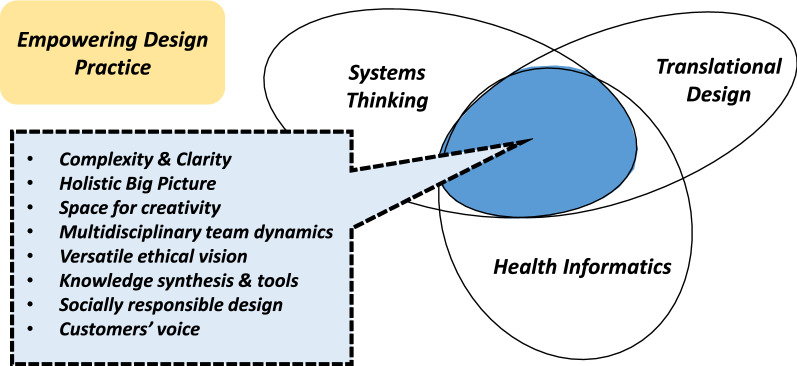
Frames supporting desing in health informatics

Certain concepts of system dynamics are reflected in translational health technology and design schemes. If design professionals could internalize such common nuances, it would ease interaction and communication between experts from different backgrounds and open important avenues in both the theoretical and practical contexts (Fig. 5). Synergies with other research and design paradigms may also be explored once information systems research matures [91]. When system complexity increases consequently the need for intense synergistic thinking and proper coordination will increase too. Novel designs require a two-way adaptation process that involves innovations and organizations, but their adaptations to consumers’ health management are of primary importance. The power of applications lies in successful adaptation. How well does the application fit its environmental entity? To what extent is it a well-functioning part of the whole? How well does it serve the targeted process entity?

## Conclusions

Synergistic thinking can enhance designers’ abilities to structure demanding design tasks and internalize the environmental whole, which is one key to well fitted applications in health informatics. It can also aid in prioritizing development choices which is necessary for cost policy reasons. As well, ethical considerations should always be deeply connected to new openings in the health sector. These abilities linked with original and open mindset aid designers in health informatics in producing more refined products.
